# Intermediate Vancomycin Susceptibility in a Community-associated MRSA Clone

**DOI:** 10.3201/eid1303.060960

**Published:** 2007-03

**Authors:** Christopher J. Graber, Margaret K. Wong, Heather A. Carleton, Françoise Perdreau-Remington, Barbara L. Haller, Henry F. Chambers

**Affiliations:** * San Francisco General Hospital, University of California, San Francisco, California, USA

**Keywords:** Staphylococcus aureus, vancomycin resistance, methicillin resistance, community-acquired infections, Panton-Valentine leukocidin, daptomycin, osteomyelitis, USA300, dispatch

## Abstract

We describe a case of treatment failure caused by a strain of USA300 community-associated methicillin-resistant *Staphylococcus aureus* (MRSA) with intermediate susceptibility to vancomycin and reduced susceptibility to daptomycin. The strain was isolated from the bone of a 56-year-old man with lumbar osteomyelitis after a 6-week treatment course of vancomycin for catheter-associated septic thrombophlebitis.

A 56-year-old man with a history of type 2 diabetes and chronic kidney disease was seen at San Francisco General Hospital in November 2005 because of hyperkalemia and volume overload. On day 4 of hospitalization, a fever of 39°C and cellulitis in the right arm associated with a peripheral intravenous line developed. Two blood cultures were drawn, the line was removed, and therapy was initiated with oral cephalexin. One of the 2 blood cultures subsequently grew methicillin-resistant *Staphylococcus aureus* (MRSA) that was susceptible to tetracycline and trimethoprim-sulfamethoxazole. The patient was treated with oral trimethoprim-sulfamethoxazole and discharged to home to complete a 10-day course. His right upper extremity cellulitis subsequently resolved, but he returned to the hospital in January 2006 with volume overload and symptoms consistent with uremia. A tunneled right internal jugular hemodialysis catheter was placed on January 11, hemodialysis was initiated, and he was discharged to home.

On February 20, he was seen in the emergency department of another facility with nausea and altered mental status. He was afebrile but hypotensive, and tenderness at the entrance site of the hemodialysis catheter was noted. Two blood cultures were positive for MRSA. The patient’s catheter was removed, and intravenous vancomycin was started. Transthoracic and subsequent transesophageal echocardiograms were negative for endocarditis, but evidence of thrombosis in the superior vena cava was seen. Multiple blood cultures were positive for MRSA through March 1, after which they became negative. The vancomycin MIC for serial isolates remained unchanged. The patient was treated with vancomycin for a 6-week course, beginning March 1. Anticoagulation with coumadin was also initiated. After clearance of his blood cultures, a right subclavian tunneled hemodialysis catheter and a left upper extremity arteriovenous fistula were placed. Vancomycin trough levels were assayed on multiple occasions during the 6-week course; all were ≥20 μg/mL.

On April 24, the patient was seen by his primary care provider for worsening bilateral knee and back pain and difficulty walking. A lumbar spine radiograph demonstrated cortical irregularity at L4-L5, indicative of discitis. He was admitted to the hospital, and vancomycin was initiated. Magnetic resonance imaging of the lumbar spine on April 25 demonstrated findings consistent with osteomyelitis and discitis at L4-L5. A needle biopsy of the L4-L5 lesion was performed on April 27. That evening, the patient was noted to be febrile and had an episode of emesis. Later in the evening, he was apneic and without a pulse, with fixed and dilated pupils. Cardiopulmonary resuscitation was performed. When neurologic function did not return during the next 4 days, supportive care was withdrawn, and the patient died May 1. Multiple blood cultures from this hospitalization remained negative, but results of a culture of lumbar fluid from a biopsy specimen on April 27 were positive for a vancomycin-intermediate *S. aureus* (VISA) isolate, with a vancomycin MIC of 8 µg/mL.

The antimicrobial susceptibility profiles of the blood isolates from November and February and the lumbar isolate from April are shown in the Table. All MIC susceptibilities were determined by broth microdilution methods per Clinical and Laboratory Standards Institute guidelines. The November and April isolates were tested with MicroScan overnight panels (Dade Behring, Deerfield, IL, USA); the February isolate was tested by using a noncommercial tray. Confirmatory testing of the vancomycin MIC of the April isolate was determined by E-test, which returned an MIC of 6 µg/mL initially and 4 µg/mL on repeat testing. The isolate also grew on vancomycin (6 µg/mL) agar screen plates. Susceptibilities to daptomycin, linezolid, and tigecycline were performed on the February and April isolates by E-test. Notably, the daptomycin MIC of the April isolate was reproducibly 2 µg/mL, an increase from 1 µg/mL for the February isolate. On blood agar plate, the April lumbar isolate was noted to be weakly β-hemolytic with small colony size. With multiple subcultures in the absence of vancomycin this morphotype reverted to the full β-hemolysis and large colony size typical of *S. aureus*, intermediate susceptibility to vancomycin was lost, and daptomycin susceptibility was regained.

**Table:** Antimicrobial susceptibility profiles of blood isolate from November 2005, blood isolate from February 2006, and lumbar isolate from April 2006*

Antimicrobial drug	MIC (μg/mL) and CLSI interpretation		
	November	February	April
Nafcillin	>2 R	16 R	>2 R
Clindamycin	2 I	≤0.25 S	<0.25 S
Erythromycin	4 I	>8 R	>4 R
Trimethoprim-sulfamethoxazole	<0.5/9.5 S	≤0.25/5 S	<0.5/9.5 S
Tetracycline	<1 S	≤0.5 S†	<1 S
Rifampin	<1 S	≤0.25 S	<1 S
Ciprofloxacin	>2 R	>4 R	>2 R
Levofloxacin	>4 R	ND	>4 R
Gentamicin	<1 S	≤0.5 S	2 S
Vancomycin	<2 S	2 S	8 I 4–6 I‡
Daptomycin	ND	1 S	2§
Linezolid	ND	2 S	2 S
Tigecycline	ND	0.125 S	0.125 S

*CLSI, Clinical and Laboratory Standards Institute; R, resistant; I, intermediately resistant; S, susceptible; ND, not done. †Susceptibility to doxycycline performed instead of to tetracycline. ‡Confirmatory susceptibility by E-test and growth on vancomycin (6 µg/mL) agar screen plates. §Interpreted as nonsusceptible by the Centers for Disease Control and Prevention. Formal CLSI breakpoints for daptomycin resistance have not been established.

Pulsed-field gel electrophoresis with *Sma*I digestion was performed on the 2 blood isolates from November and February and the lumbar isolate from April. All 3 isolates shared an identical pattern with the USA300-0114 control strain (Figure). Sequencing of the protein A gene polymorphic region (*spa* typing) (*1*) of the lumbar isolate obtained the sequence YHGFMBQBLO, typical for clonal cluster 8. PCR was positive for the presence of *mecA*, ACME (arginine catabolic mobile element, a signature gene cluster of USA300), and Panton-Valentine leukocidin genes and negative for the presence of the *vanA* gene, when primers and sequences previously described were used (*2*–*4*).

**Figure:**
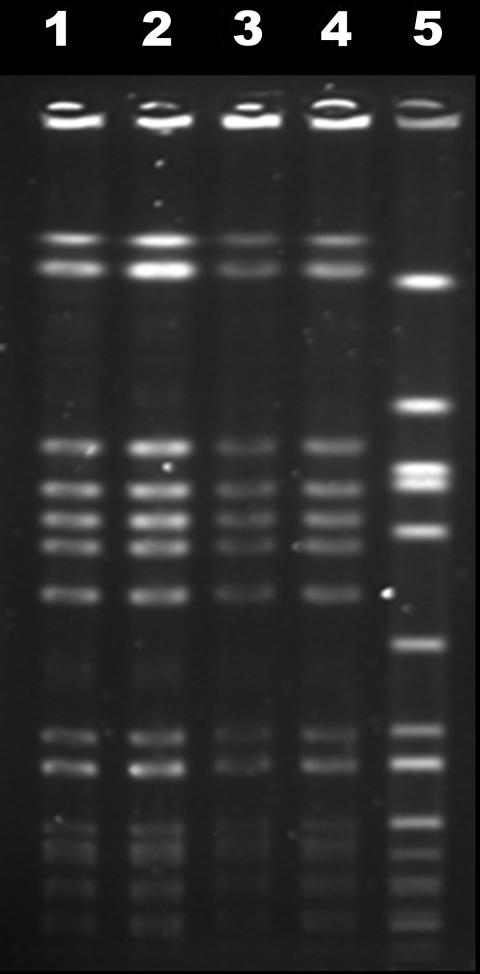
Pulsed-field gel electrophoresis profiles of November and February blood isolates (lanes 1 and 2), April lumbar isolate (lane 3), reference USA300-0114 isolate (lane 4), and internal control g195a (lane 5).

The community MRSA clone USA300, first identified in 2001, has emerged as a notable cause of colonization and disease in San Francisco (*5*). This clone has been remarkable for its rapid spread and propensity to cause severe infection, particularly skin and soft tissue and pulmonary infection. Recent sequencing of its genome demonstrated possible contributors to its virulence, most notably Panton-Valentine leukocidin and ACME (*2*). The USA300-0114 subclone has been the predominant one during the community MRSA epidemic in San Francisco (*6*) and is becoming widely prevalent in communities throughout the United States (*7*). Surveys of clinical MRSA isolates throughout the city of San Francisco have shown an explosive increase in the prevalence of disease caused by USA300 and displacement of other clones in both inpatient and outpatient settings since 2001, blurring the clone’s distinction as community associated.

The first report of clinical VISA infection was from Japan in 1997 (*8*). Molecular typing of this and subsequent VISA isolates showed these to be derived from prevalent hospital-acquired clones primarily belonging to clonal cluster 5 (*9*). The accessory gene regulator (*agr*) group II genotype present in clonal cluster 5 strains has been speculated to predispose to emergence of the VISA phenotype. USA300 is an *agr* group III clone (*6*). The occurrence of the vancomycin-intermediate phenotype in USA300 suggests that development of this phenotype may simply reflect the prevalence of clones in a particular population, rather than a causal relationship to *agr*.

The mechanism of reduced vancomycin susceptibility in VISA is thought to be mediated by an increase in the number of false targets because of a thickened cell wall (*10*), perhaps aided by altered expression of penicillin-binding proteins 2 and 4 (*11*). VISA and hVISA, strains of *S. aureus* that contain subpopulations of daughter cells displaying intermediate sensitivity to vancomycin but for which the MICs for vancomycin fall within the susceptible range, can be difficult to detect in the microbiology laboratory because the phenotypes are unstable and can be lost on subsequent passages (*12*); this situation was demonstrated in our case. Reduced susceptibility to daptomycin in vancomycin-intermediate isolates has been described previously, perhaps because of reduced diffusion of the molecule through the thickened cell wall, although this has not been proven (*13*). The clinical importance of the reduced daptomycin susceptibility seen in vancomycin-intermediate isolates, however, is unclear at this time.

Our patient experienced clinical failure of vancomycin therapy despite high serum drug levels, which speaks to the difficulty with which highly invasive *S. aureus* infections are successfully treated with vancomycin, particularly in patients receiving hemodialysis. While most VISA isolates reported in the United States have been isolated from patients receiving hemodialysis, chronic renal disease and hemodialysis have not been definitively identified as risk factors for infections caused by VISA or hVISA (*14*). The frequency of nasal colonization with hVISA was low in hemodialysis patients monitored from 1999 to 2002 in the San Francisco Bay Area (*15*). However, as the prevalence of USA300 increases and prompts further use of vancomycin, intermediate vancomycin susceptibility in USA300 may become more common among both community and hospital isolates.
